# *Anopheles gambiae* salivary protein expression modulated by wild *Plasmodium falciparum* infection: highlighting of new antigenic peptides as candidates of *An. gambiae* bites

**DOI:** 10.1186/s13071-014-0599-y

**Published:** 2014-12-20

**Authors:** Alexandra Marie, Philippe Holzmuller, Majoline T Tchioffo, Marie Rossignol, Edith Demettre, Martial Seveno, Vincent Corbel, Parfait Awono-Ambéné, Isabelle Morlais, Franck Remoue, Sylvie Cornelie

**Affiliations:** MIVEGEC (UMR IRD224 CNRS 5290 UM1-UM2), Institut de Recherche pour le développement (IRD), 911 avenue Agropolis, Montpellier cedex 5, 34394 France; CIRAD Département Systèmes Biologiques BIOS UMR 15 CMAEE “Contrôle des Maladies Exotiques et Emergentes”, Campus International de Baillarguet, TA A-15/G, Montpellier cedex 5, 34398 France; Institut de Génomique Fonctionnelle, CNRS UMR 5203, INSERM U661, UM1, UM2, Plate-forme de Protéomique Fonctionnelle CNRS UMS BioCampus 3426, Montpellier, 34094 France; Department of Entomology, Faculty of Agriculture, Kasetsart University, 50 Ngam Wong Wan Rd, Ladyaow Chatuchak, Bangkok, 10900 Thailand; Laboratoire de Recherche sur le Paludisme, Organisation de Coordination pour la lutte contre les Endémies en Afrique Centrale (OCEAC), Yaoundé, BP 288 Cameroun; MIVEGEC- IRD- CREC, Cotonou, 01 BP4414 RP Bénin

**Keywords:** *Anopheles gambiae*, Wild *Plasmodium falciparum*, Salivary proteins, Biomarker, Infective bites, Proteomic

## Abstract

**Background:**

Malaria is the major parasitic disease worldwide caused by *Plasmodium* infection. The objective of integrated malaria control programs is to decrease malaria transmission, which needs specific tools to be accurately assessed. In areas where the transmission is low or has been substantially reduced, new complementary tools have to be developed to improve surveillance. A recent approach, based on the human antibody response to *Anopheles* salivary proteins, has been shown to be efficient in evaluating human exposure to *Anopheles* bites. The aim of the present study was to identify new *An. gambiae* salivary proteins as potential candidate biomarkers of human exposure to *P. falciparum*-infective bites.

**Methods:**

Experimental infections of *An. gambiae* by wild *P. falciparum* were carried out in semi-field conditions. Then a proteomic approach, combining 2D-DIGE and mass spectrometry, was used to identify the overexpressed salivary proteins in infected salivary glands compared to uninfected *An. gambiae* controls. Subsequently, a peptide design of each potential candidate was performed *in silico* and their antigenicity was tested by an epitope-mapping technique using blood from individuals exposed to *Anopheles* bites.

**Results:**

Five salivary proteins (gSG6, gSG1b, TRIO, SG5 and long form D7) were overexpressed in the infected salivary glands. Eighteen peptides were designed from these proteins and were found antigenic in children exposed to the *Anopheles* bites. Moreover, the results showed that the presence of wild *P. falciparum* in salivary glands modulates the expression of several salivary proteins and also appeared to induce post-translational modifications.

**Conclusions:**

This study is, to our knowledge, the first that compares the sialome of *An. gambiae* both infected and not infected by wild *P. falciparum*, making it possible to mimic the natural conditions of infection. This is a first step toward a better understanding of the close interactions between the parasite and the salivary gland of mosquitoes. In addition, these results open the way to define biomarkers of infective bites of *Anopheles*, which could, in the future, improve the estimation of malaria transmission and the evaluation of malaria vector control tools.

**Electronic supplementary material:**

The online version of this article (doi:10.1186/s13071-014-0599-y) contains supplementary material, which is available to authorized users.

## Background

In Sub-Saharan Africa, *Anopheles gambiae* is the main vector of *Plasmodium falciparum*, the most deadly of the five human *Plasmodium* species, responsible for malaria. Over half a million deaths (627,000) occur every year, especially in children under 5 years of age, according to WHO [1]. Due to the lack of vaccines, the spread of resistance to anti-malaria drugs [2] and the difficulty in accessing drug treatment (the artemisinin-based combination therapies), vector control using Long Lasting Insecticide-treated Nets (LLIN) and/or Indoor Residual Spraying (IRS) still remains an important component of malaria prevention and control. However, the development of insecticide resistance in the main malaria vectors in Africa [3] is challenging the success of malaria vector control strategies [4,5]. In a context of malaria elimination in some areas, integrated malaria control campaigns have been implemented to reduce the malaria burden. Consequently, in these areas where transmission has substantially decreased, but also in urban settings or high-altitude areas where *Anopheles* exposure and malaria transmission can be very low, the current methods are not sufficiently sensitive to evaluate the human exposure to *Anopheles* bites and the risk of transmission. Indeed it appears difficult to obtain precise information on parasite detection and mosquito capture in these contexts so the development of appropriate tools is necessary.

One promising approach is to evaluate the real contact between the human host and the vector by measuring the human antibody (Ab) response to specific *Anopheles* salivary proteins [6]. During its blood meal, mosquitoes inject saliva into the human skin, inducing a humoral response. This concept has been validated using whole saliva extracts (WSEs) of *An. gambiae* [6] and in other hematophagous arthropods, such as *Aedes* [7-9], *Culex* [10,11], *Glossina* [12,13] and phlebotomine sand flies [14,15]. However, some salivary proteins are ubiquitous in arthropods and the response observed against WSEs could therefore reflect the exposure to numerous arthropods. For this reason, a biomarker of human exposure to bites must be directed to genus- or species-specific epitopes. Based on previous studies, the gSG6 protein has been shown to be specific to the *Anopheles* genus and immunogenic [16,17]. This protein was therefore validated as a specific biomarker of exposure to *Anopheles* bites in Burkina Faso [18,19] and Tanzania [20].

To optimize the specificity and the utility of the biomarker, a peptide design of this protein was performed. The gSG6-P1 peptide has been found to be antigenic and the Ab response to this peptide was positively associated with the level of exposure to *Anopheles* bites [21]. This peptide has also been validated as a biomarker in different malaria transmission areas such as rural low exposure in Senegal [22,23], in highland areas in Kenya [24], in urban settings [25] and for exposure to *An. funestus* [26]. The limitation of this biomarker is that the Ab response to the gSG6 protein and the gSG6-P1 peptide may not discriminate between infective and non-infective bites, hence limiting the estimation of malaria transmission intensity. Settings of malaria transmission could be very different depending on field conditions, from unstable to stable malaria, with sporozoite rates ranging from 0.1% to 8% [27,28]. In low-transmission areas, the exposure to all *Anopheles* bites does not accurately represent the malaria transmission risk [29]. Moreover, hotspots of malaria transmission exist in all epidemiological settings, maintaining transmission in low-transmission seasons and fueling transmission in high-transmission seasons; the detection of these hotspots will make it possible to concentrate the integrated malaria controls [30]. Consequently, a new biomarker specific to infective bites has to be developed to assess precisely malaria risk in these particular settings and could also be useful for evaluating the efficacy of malaria control tools (drug treatments and vector controls).

The salivary glands are the crucial organ for the development and transmission of the parasite to a host. *Plasmodium* interacts with salivary proteins to enter salivary glands [31-34]. To survive and multiply in this organ, parasites have to counteract the immune system and use the vector’s metabolism by modifying salivary protein expression [35]. Saliva is also essential for the success of blood-feeding [36], and consequently, the modification of salivary protein expression could promote parasite development and transmission. Rossignol et al., have demonstrated for the first time that the *Plasmodium* infection in mosquitoes decreased the expression of a salivary protein, the apyrase enzyme [37]. Transcriptomic studies have shown that genes encoding for salivary proteins were up- or down-regulated in *Ixodes scapularis* nymphs infected by flavivirus [38], *Rhipicephalus microplus* infected by *Anaplasma marginale* [39], *Culex quinquefasciatus* infected by West Nile virus [40] and *Aedes aegypti* infected by different serotypes of dengue virus (DENV) [41]. Proteomic analyses have indicated that salivary proteins were modulated in *Glossina pallipides* infected by salivary gland hypertrophy virus [42], *Ae. aegypti* infected by DENV serotype 2 (DENV-2) [43], *Ae. albopictus* infected by DENV-2 [44] and *Ae. aegypti* infected by chikungunya virus (CHIKV) [45]. As for *Plasmodium* parasites, several studies have investigated the change of salivary protein expression in *An. gambiae* and *An. stephensi* infected by murine *Plasmodium* [46-49]. The iTRAq technique, used by Choumet et al., showed that the expression of five salivary proteins was modulated, with the ratio between infected *versus* non-infected salivary gland varying from 0.65 to 1.97 [46]. In a more recent study using the 2D-PAGE method, seven salivary proteins were shown with a modulated expression in the presence of *Plasmodium berghei* varying from 0.28 to 12 [47]. The present study aimed to identify salivary proteins as potential biomarkers of *An. gambiae* infective bites. To mimic field conditions and thereby evaluate salivary protein modulation as accurately as possible, experiments were conducted in semi-field conditions. *An. gambiae* mosquitoes were fed through membrane on blood containing *P. falciparum* gametocytes from naturally infected donors. Comparison of sialome in *P. falciparum*-infected *versus* non-infected salivary glands of *An. gambiae* was achieved by 2D-Differential Gel Electrophoresis (2D-DIGE) and mass spectrometry (LC-MS/MS). Peptides were designed from protein candidates and their immunogenicity was tested in sera from humans living in malaria areas. These methods allowed us to select immunogenic peptides which could represent potential candidate biomarkers of *An. gambiae* infective bites.

## Methods

### Ethics statements

Experimental infections involving human subjects were approved by the Cameroonian National Ethical Committee (statement 099/CNE/SE/09). Children identified as gametocyte carriers were enrolled as volunteers after their parents or legal guardians had signed an informed consent form. Collection of human blood on filter papers was approved by the National Ethics Committee of the Senegal Ministry of Health (October 2008; 0084/MSP/DS/CNRS, ClinicalTrials.gov ID: NCT01545115). Oral and written informed consent was obtained from the children’s parents or legal guardians.

### Mosquito strain

The Kisumu strain of *An. gambiae* mosquitoes was reared in the insectary at the OCEAC (Yaounde, Cameroon). Adult mosquitoes were maintained in controlled conditions (27 ± 2°C, 85 ± 5% RH, and 12 h light/dark) and provided with a 6% sterile sucrose solution.

### Experimental infections

Female mosquitoes were fed on *P. falciparum* gametocyte carriers. Infectious feeding was conducted as previously described [50,51]. Females, 3–5 days old, were starved for 24 h and allowed to feed on blood containing *P. falciparum* gametocytes (from 52.7 to 60.6 gametocytes/μL) for 35 min. Non-infected salivary glands were obtained by feeding female mosquitoes on the blood from the same donors but heated at 43°C for 12 min for gametocyte inactivation [52]. Unfed and partially fed mosquitoes were removed by aspiration and discarded. Fully engorged females were kept in the insectary until dissections 14 days after the infectious blood meal. Salivary glands were dissected in buffer containing Urea, 7M; Thiourea, 2M; CHAPS, 4%. Samples were frozen individually until processing.

### Protein sample preparation

Infected and non-infected salivary glands were lysed in liquid nitrogen and homogenates were then centrifuged for 20 min at 30,000 × g at 17°C. The supernatants, called salivary gland extracts (SGEs), were collected, purified using a 2D Cleanup Kit (GE Healthcare) and protein concentrations were measured using a Coomassie Plus Protein kit (Pierce). SGEs from about 75 salivary glands were pooled to obtain 15 μg of protein for each batch.

### 2D Differential Gel Electrophoresis (2D-DIGE)

*P. falciparum*-infected and non-infected protein samples were compared using the CyDye DIGE Fluors for Ettan DIGE (GE Healthcare, Germany) for four gel replicates.

15 μg of SGE from *P. falciparum*-infected and non-infected samples was labeled with 150 pmol/μL of either Cy3 or Cy5 following the manufacturer’s recommendations. An internal standard comprising 7.5 μg of each SGE was labeled with Cy2. A dye swap was performed to ensure that the modifications observed between the two conditions were not due to different efficiencies in dye labeling. IEF was performed with 11 cm Immobiline DryStrip, pH 3–11 nonlinear (NL) (GE Healthcare, Germany). The run conditions were: current 50 μA per strip; 60 V (step) for 1 h, 1000 V (gradient) for 2 h, 6000 V (gradient) for 2 h and then 6000 V steps up to 30,000 Vh. The second dimension was carried out on 10–20% SDS-PAGE gels (Biorad, Marnes-la-Coquette, France) at 30 V for 20 min and then 200 V until the bromophenol blue front had reached the bottom of the gel. Gels were scanned using a Typhoon 9400 imager (GE Healthcare, Germany). Images were acquired at 100 μm pixel resolution under non-saturating conditions and were analyzed with Progenesis Samespots 3.3 software. Statistical analysis and protein quantification were performed with ANOVA test (p < 0.005), which took into account the mean difference and the variance between the infected and non-infected groups. The fold change with a cut-off of 1.4-fold over- and under-expression was used. The statistical power of this study was greater than 0.9.

### Protein identification by LC-MS/MS

For the spot excision, gels were stained with the PageBlue Protein Staining Solution (Fermentas).

### Trypsin digestion

Enzymatic in-gel digestion was performed according to the Shevchenko modified protocol [53]. Briefly, gel slices were destained by three washes in 50% acetonitrile, 50 mM triethylammonium bicarbonate buffer and incubated overnight at 25°C (with shaking) with 300 ng trypsin (Gold, Promega, Charbonnières, France) in 100 mM triethylammonium bicarbonate buffer. Tryptic peptides were extracted with 50% acetonitrile and 5% formic acid, and dehydrated in a vacuum centrifuge.

### Nano LC-MS/MS analysis

Peptides were solubilized in 2 μL of 0.1% formic acid - 2% acetonitrile and analyzed online by nano-flow HPLC-nanoelectrospray ionization using a LTQ Orbitrap XL mass spectrometer (LTQ Orbitrap XL, Thermo Fisher Scientific, San Jose, CA, USA) coupled with an Ultimate 3000 HPLC (Dionex). Desalting and pre-concentration of samples were performed on-line on a Pepmap® precolumn (0.3 mm × 10 mm). A gradient consisting of 0-40% B for 30 min, 80% B for 15 min (A = 0.1% formic acid, 2% acetonitrile in water; B = 0.1% formic acid in acetonitrile) at 300 nL/min was used to elute peptides from the capillary (0.075 mm × 150 mm) reverse-phase column (Pepmap®, Dionex) fitted with an uncoated silica PicoTip Emitter (New Ojective, Woburn, MA, USA). LC-MS/MS experiments comprised cycles of five events; an MS1 scan with Orbitrap mass analysis at 60,000 resolutions followed by collision induced dissociation (CID) of the five most abundant precursors. Fragment ions generated by CID were detected at the linear trap. Normalized collision energy of 35 eV and activation time of 30 ms were used for CID. All Spectra were recorded under positive ion mode using the Xcalibur 2.0.7 software (Thermo Fisher Scientific). Spectra were acquired with the instrument operating in the information-dependent acquisition mode throughout the HPLC gradient. The mass scanning range was m/z 400–2000 and standard mass spectrometric conditions for all experiments were: spray voltage, 2.2 kV; no sheath and auxiliary gas flow; heated capillary temperature, 200°C; capillary voltage, 40 V and tube lens, 120 V. For all full-scan measurements with the Orbitrap detector, a lock-mass ion from ambient air (m/z 445.120024) was used as an internal calibrant as described [54].

All MS/MS spectra were searched for against the Insecta entries of either SwissProt or TrEMBL databases (http://www.uniprot.org/; v 2012_07) using the Proteome Discover software v 1.3 (Thermo Fisher Scientific) and Mascot v 2.3 algorithm (http://www.matrixscience.com/) with trypsin enzyme specificity and one trypsin missed cleavage. Carbamidomethylation was set as fixed cystein modification and oxidation was set as variable methionine modification for searches. A peptide mass tolerance or 5 ppm and a fragment mass tolerance of 0.5 Da were allowed for identification.

Management and validation of mass spectrometry data were carried out using Proteome Discoverer software v 1.3 (p < 0.01 for 2 peptides or more/protein).

### Peptide design

The design of potential immunogenic peptides was investigated using an *in silico* approach. The putative B-cell epitopes were identified with the BcePred [55], ABCpred [56], BepiPred [57] and SVMTrip databases [58]. The sequences were aligned with the Blastp program in the Vectorbase database [59] and the UniProtKB database to compare the peptide sequences with known genomes or EST libraries. The peptides were selected when at least three to four algorithms predicted the same epitopes.

### Peptide array

Experiments were performed with EpiFlag® methodology (Innobiochips, Lille, France). Eighteen peptides of 18–27 amino acids were synthesized by solid-phase peptide synthesis with an automated peptide synthesizer (Intavis AG, Köln, Germany) using the Fmoc/tert-butyl strategy on a 20 μmol scale on a Rink-ChemMatrix® (PCAS BioMatrix Inc, Saint-Jean-sur-Richelieu, Quebec, Canada) resin. Following their elongation, peptides were deprotected and cleaved for 3 h at room temperature (RT) using TFA/water/triisopropylsilane/EDT (1850 μL/50 μL/50 μL/50 μL), precipitated in diethyl ether/n-heptane, 1/1 v/v, purified by RP-HPLC on a 120-Å, 5 μm C18 Nucleosil column using a linear water/acetonitrile gradient containing 0.05% TFA by volume (6 mL/min, detection at 215 nm) and lyophilized.

Each peptide characterized by RP-HPLC and MALDI-TOF MS, was dissolved to a final concentration of 0.1 mM in 0.01 M PBS, pH 7.4, and printed on amine-modified glass slides (Arrayit, Sunnyvale, CA, USA) in duplicate.

Peptide arrays were blocked for 1 h at RT with PBS-M (0.01 M PBS, pH7.4, 0.05% Tween 20 and 2.5% non-fat milk). Saturated microarrays were washed with PBS containing 0.05% Tween 20. Human sera from Senegalese children infected by *P. falciparum* or not and exposed to *Anopheles* bites, were diluted 1:10 in PBS-M and incubated overnight at 4°C. Microarrays were then washed three times with PBS containing 0.05% Tween 20. After washing, the microarrays were revealed using a AlexaFluor 555-labeled goat polyclonal anti-human IgG antibody (Life Technology, Saint Aubin, France) at 1 μg/mL in PBS-M, for 1 h at RT. Microarrays were washed, rinsed with distilled water and dried. The glass slides were scanned with a TECAN LS-reloaded scanner (Tecan, Männedorf, Switzerland): PMT = 150. Data were extracted using Array-Pro® Analyzer Software.

## Results and discussion

### Difference in sialome profile between *P. falciparum*-infected and non-infected salivary glands of *An. gambiae*

**Figure 1:**
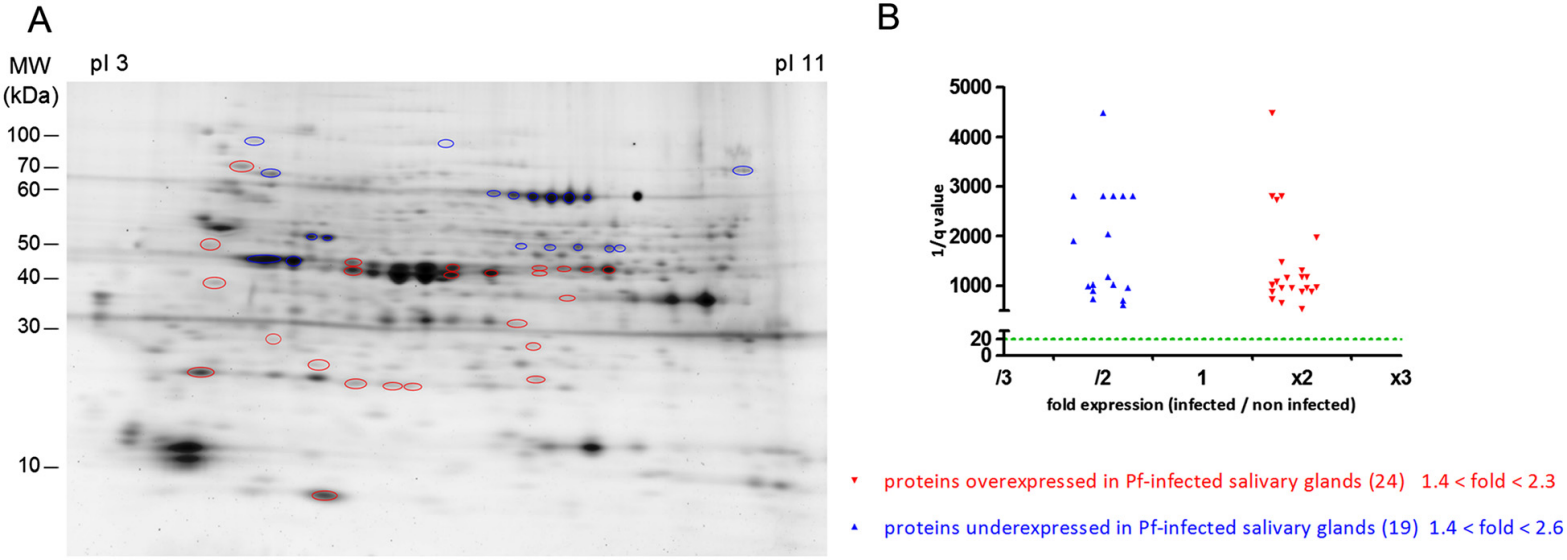
**Differential salivary protein expression of** ***An. gambiae*** **infected or not infected by wild** ***P. falciparum*** **. (A)** 2D-DIGE gel. Forty-three differentially expressed spots, and the only spots which have been excised, are indicated by circles. Red circles represent the 24 over-expressed spots and the 19 blue circles represent the under-expressed spots. Proteins were separated in the first dimension using carrier ampholyte gradient gels between pH 3 and pH 11. The second dimension was separated using on 10–20% SDS-PAGE gels. The isoelectric point (pI) and molecular weight scales are indicated in the figure. **(B)** Differences of protein expression are represented according to the expression ratio (infected/non-infected) and significance ratio (q value). The horizontal dotted line indicates the significance threshold of q < 0.05 (or 1/q > 20) according to the Samespot analysis.

The salivary glands were dissected 14 days post-infection, i.e. the time period needed for the parasites to reach the salivary glands. The infectious status of each pair of salivary glands was checked by quantitative PCR [60]. The differential expression of the sialome between *P. falciparum*-infected and non-infected salivary glands of *An. gambiae* was assessed using 2D-DIGE. Overall, four biological replicates of gel were performed. After the ANOVA analysis and adjustment using the FDR approach, 207 spots showed a significant differential profile (q < 0.01 and power >0.9) with a modulation from 1.3- to 8.8-fold. Among them, 128 spots were over-expressed and 79 under-expressed. After colloidal Coomassie blue staining, 43 visible spots could be excised for LC-MS/MS identification (Figure 1A), which represent less than one third of the regulated spots and constitute an additional constraint to the identification of relevant biomarkers. Among them, 24 spots presented a 1.4- to 2.3-fold over-expression in *P. falciparum*-infected salivary glands, whereas 19 spots presented a 1.4- to 2.6-fold under-expression (Figure 1B). These fold changes were in accordance with another study comparing the modification of the *An. gambiae* sialome infected by *P. berghei* using the iTRAQ technology, in which the modulations are within the same range (from 1.5 to 1.95). However, in this previous study, the expression of only five proteins was found to be altered [46]. Another study using the *An. gambiae*-*P. berghei* experimental model has shown that the expression of seven salivary proteins was changed from 3.5- to 12-fold with the 2D-PAGE technique [47]. The larger number of differentially expressed spots in the present experiment may be due to broader protein regulation upon *P. falciparum* infection or alternatively to the different techniques and protocols used between studies.

### Identification of overexpressed proteins in *P. falciparum*-infected *An. gambiae* salivary glands

**Figure 2:**
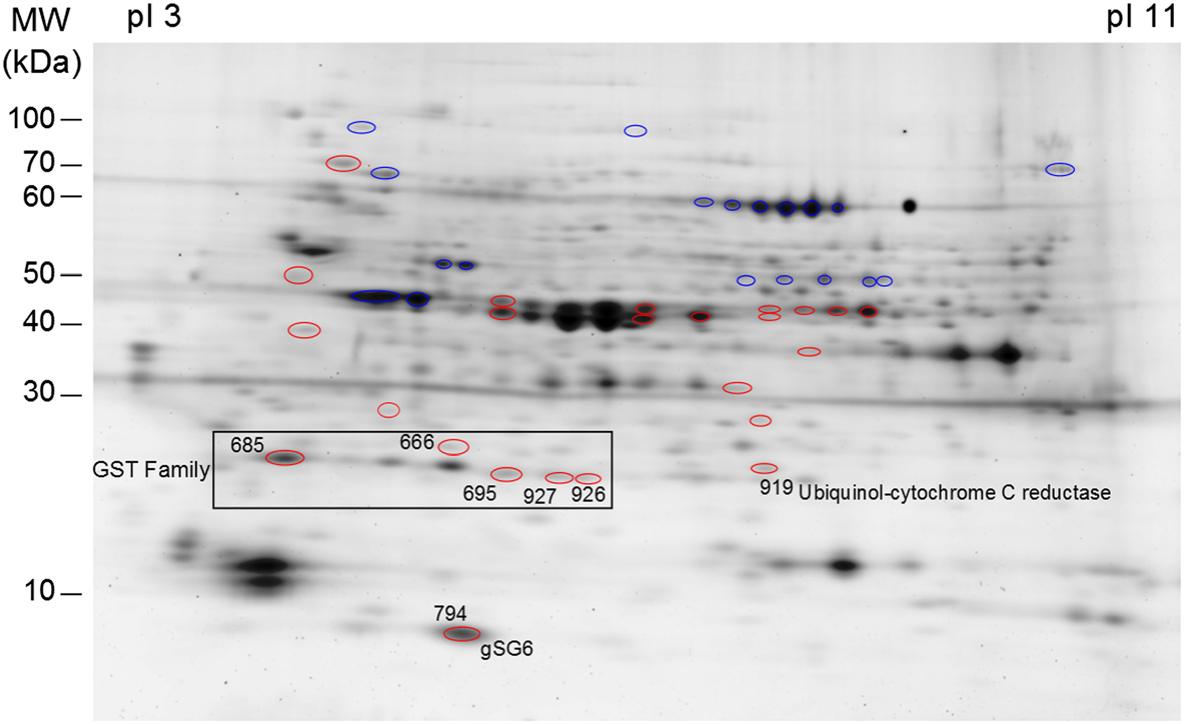
**Overexpressed proteins in** ***P. falciparum*** **-infected** ***An. gambiae*** **salivary glands (2D-DIGE gel).** Over-expressed protein spots are indicated by number. The pI and molecular weight scales are indicated in the figure.

Among the 43 overexpressed spots, two proteins, the ubiquinol cytochrome c reductase iron-sulfur subunit and the gSG6 were found and identified, each in a single spot, as a unique protein (Figure 2 and Table 1).

**Table 1:** **Upregulated proteins in** ***P. falciparum*** **-infected** ***An. gambiae*** **salivary glands**

**Spot**	**Accession number (UniProtKB/TrEMBL)**	**Protein identification**	**Fold**	**Molecular mass (kDA)**	**pI**	**Mascot score**	**Sequence coverage (%)**
666	P46428_ANOGA	GST S1	2.3	23.2	5.29	7	29.06
	Q9GPL9_ANOGA	GST E1		25.3	5.66	4	17.86
685	P46428_ANOGA	GST S1	2.2	23.2	5.29	9	33.99
695	Q93113_ANOGA	GST D1 iso D	2.0	23.4	6.34	5	25.36
	Q93112_ANOGA	GST D1 iso C		23.8	6.55	3	20.57
927	Q93112_ANOGA	GST D1 iso C	2.3	23.8	6.55	8	36.36
926	Q93112_ANOGA	GST D1 iso C	2.0	23.8	6.55	5	17.70
919	Q7PWI1_ANOGA	Ubiquinol cyt c reductase iron sulfur subunit	2.0	28.1	8.53	3	9.43
794	Q9BIH5_ANOGA	gSG6	1.8	13.1	5.49	14	45.22

The ubiquinol-cytochrome c reductase iron-sulfur subunit (Rieske subunit) is an essential component of complex III implicated in the oxidative phosphorylation. The increase in the expression of this enzyme involved in the oxidative phosphorylation has already been observed in the head of *An. gambiae* infected by *P. berghei* [61]. The presence of *P. falciparum* in *An. gambiae* could thus increase oxidative metabolism, which could be a response involved in parasite resistance.

The gSG6 protein was first identified in *An. gambiae* [62] but also found in *An. stephensi* [16] and *An. funestus* [63] and is specifically expressed in salivary glands in female mosquitoes [64]. This protein has not been found in *Culex* [65] and *Aedes* [66,67] mosquitoes, suggesting that it is specific to *Anophelinae* mosquitoes. A study has demonstrated that this protein plays a role in blood feeding [68]. In the present study, the infection of salivary glands of *An. gambiae* by wild *P. falciparum* induced over-expression of the gSG6 protein (+1.8-fold). However, other proteomic studies in murine models of malaria have shown that the gSG6 protein was down-regulated in *An. gambiae* infected by *P. berghei* [46,47]. Discrepancies between studies may result from exposure to different strains of parasites. It is known that a strong relationship between pathogens and their hosts exists, which generates mutual co-evolution and co-adaptation [52,69,70]. In our study, we used the natural model of human malaria, *An. gambiae* and *P. falciparum*, whereas in the other studies *An. gambiae* was infected by a rodent malaria parasite *P. berghei*, whose natural mosquito vector is *An. dureni* [71]. In addition, we used natural isolates of *P. falciparum* and parasite transmission efficiency relies on multiple factors, such as density, sex ratio or genetic complexity [52,72-74]. Interestingly, gene regulation upon *P. falciparum* infection in the mosquito midgut is infection intensity-dependent [52]. In the present study, the gametocyte loads in infected individuals ranged from 52.7 to 60.6 gametocytes/μL of blood, which is low compared to parasite densities used with *P. berghei* in laboratory conditions. Differences in parasite densities within salivary glands could also lead to different levels of protein expression in the two parasite species.

In the present study, several proteins belonging to the glutathione S-transferase (GST) family were also upregulated: GST S1, GST E1, GST D1 isoform D and GST D1 isoform C. GST S1 and GST D1 isoform C were identified in several closed spots (Figure 2 and Table 1). This could be due to post-translational modifications such as glycosylation, phosphorylation and acetylation. The GST proteins are strongly involved in diverse biological processes in almost organisms such as detoxification of endogenous and xenobiotic compounds as well as in protein transport and protection against oxidative stress [75,76]. This protein family is conserved in the majority of arthropods such as *Ae. aegypti* [77] and *Cx. quiquefasciatus* [78]. The delta and epsilon classes of GST are insect-specific [79]. The GST D1 isoform C and D belongs to the delta class. These genes are rapidly diverging, suggesting a role in the adaptation of insects in different ecological niches and may be involved in the detoxification of environmental xenobiotics. This hypothesis is supported by the implication of this delta class in insecticide resistance [80]. GST E1 belongs to the epsilon class implicated in the detoxification of insecticides and in the resistance to DDT [81-83]. They also have peroxidase activity, which could be involved in the protection against the secondary effects of oxidative stress [84]. The GST S1 protein belongs to the sigma class. This class of GST is found in indirect flight muscles, suggesting a structural role. However, it could also protect against the deleterious effects produced by oxidative stress [76]. It has been shown that the malaria infection in mosquito midguts induced an oxidative stress producing reactive oxygen species [85]. Consequently, the increase of GST family proteins probably counteracts the negative effects induced by the infection.

For the other spots, the identification of up- or down-regulated proteins was more complex. Numerous proteins were identified in each spot, which did not allow us to determine which proteins have their expression modified (data not shown). Moreover, several proteins were found both in up- and down-regulated spots, certainly due to post-translational modifications, which made it impossible to conclude whether their expression had been modulated. However, some identified proteins in overexpressed spots involved in a glycolysis pathway (triosephosphate isomerase, fructose biphosphate aldolase, phosphoglycerate mutase and glyceraldehydes-3-phosphate dehydrogenase) were also found upregulated during DENV-3 infection in the cell line of *Ae. albopictus* [86], DENV-2 or CHIKV-infected midgut of *Ae. aegypti* [45] as well as in the *P. berghei*-infected head of *An. gambiae* [61]. In addition, proteins involved in lipid metabolism were found in overexpressed spots, in agreement with a previous transcriptomic study [48]. All these studies strengthen our hypothesis that these identified proteins are probably upregulated. Moreover, these metabolic pathways are involved in energy production, which is in accordance with the over-expression of the ubiquinol-cytochrome c reductase iron-sulfur subunit. *P. falciparum* infection seems to interfere with the metabolic processes of *An. gambiae* salivary glands. This has already been observed during the influenza virus [87] and *Leishmania* [88] infection.

Surprisingly, only seven *P. falciparum* proteins were found in over- and under-expressed spots (Additional file 1). This result could be due to the protein extraction protocol being insufficiently severe to disrupt sporozoites. We can also assume that the amount of salivary gland proteins was much greater resulting in a high salivary gland protein to parasite protein ratio, thus precluding the detection of the *P. falciparum* proteins.

### Identification/selection of candidate biomarkers of exposure to *Anopheles* infective bites

The selection of candidates as specific biomarkers for infective bites was based on several criteria: i) over-expression, i.e. overexpressed proteins or proteins found in over-expressed spots with a high percentage of sequence coverage and a high number of identified peptides, suggesting that they are the major component of the spot; ii) potential antigenicity, i.e. inferred from the presence of a signal peptide in the protein sequence, meaning that they are secreted in the saliva and injected into the human skin during blood feeding and, as a consequence potentially induce an Ab response in humans; and iii) the specificity of proteins to the *Anopheles* genus. As the salivary protein candidates are overexpressed, one hypothesis could be that their antigenicity is increased, only after *P. falciparum* infection, allowing the differentiation between infective and non-infective bites. Among the overexpressed proteins mentioned above, the ubiquinol-cytochrome c reductase Rieske subunit and the GST proteins are common in many organisms and are not secreted proteins. Consequently, they are not suitable candidates for being a specific biomarker to *Anopheles* infected bites.

The gSG6 protein has been previously shown to be a biomarker of exposure to *Anopheles* bites [17-20] and some peptides have also been designed from this protein. Among them, gSG6-P1 and gSG6-P2 peptides were antigenic, but only the gSG6-P1 peptide seemed positively associated with the level of human exposure to *Anopheles* [21], in different exposure settings [22-25]. In the present study, the expression of the gSG6 protein was clearly increased in presence of wild *P. falciparum*. This result suggests that this protein could also be a potential candidate as a biomarker of infective bites. Recent studies strengthen this point by demonstrating that the Ab response to the gSG6 recombinant protein was associated with malaria incidence in Tanzania [20] and that the gSG6-P1 peptide could be an indicator of infection risk during the dry season (very low exposure and transmission) in northern Senegal [89].

**Figure 3:**
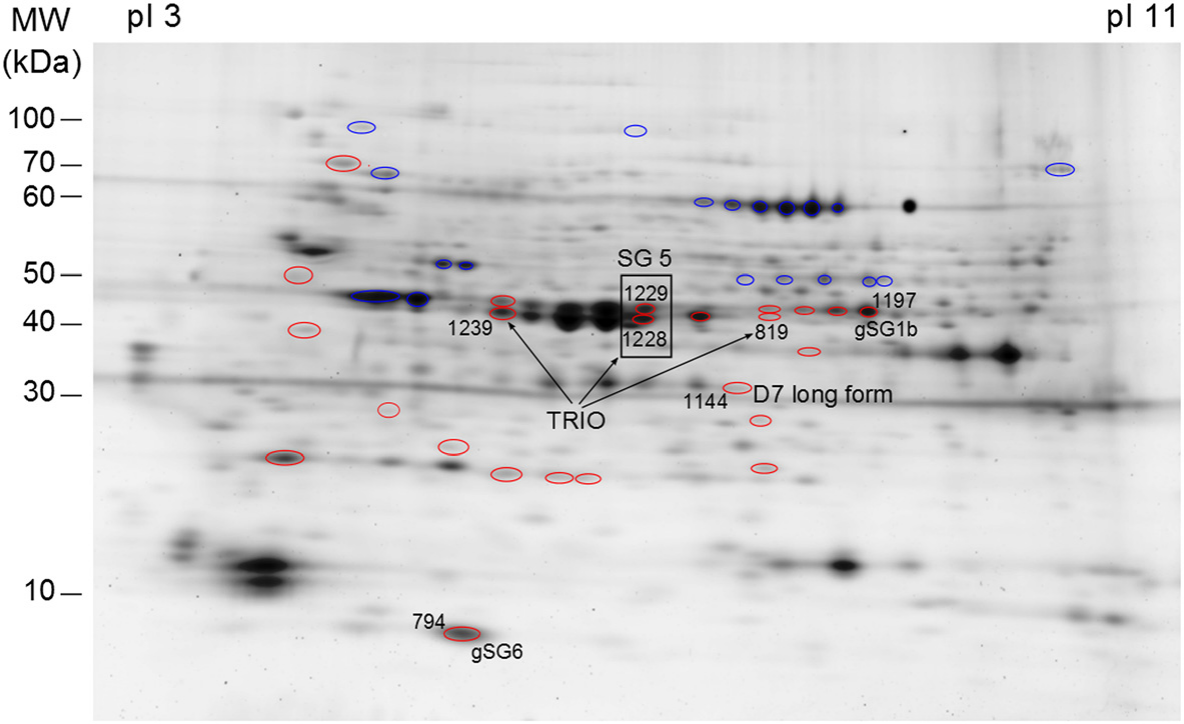
**Candidate proteins as biomarkers of** ***Anopheles*** **infective bites (2D-DIGE gel).** Spots containing candidate proteins are indicated by number. The pI and molecular weight scales are indicated in the figure.

Other candidate proteins as biomarkers were selected from some over-expressed spots: gSG1b, TRIO protein, long form D7 and SG5 (Figure 3 and Table 2). The gSG1b and TRIO proteins have already been found to be overexpressed in *P. berghei*-infected salivary glands of *An. gambiae* [47]. This result supports the finding that, in the present study, these proteins, identified among others in overexpressed spots, seem clearly upregulated. The long form D7 and SG5 proteins were selected, although their sequences matched other arthropods but with a low identity. The long form D7 protein presented 35.1% identity with *Ae. Aegypti* (e = 8e^−51^), 34.1% with *Cx. quiquefasciatus* (e = 5e^−53^), 31.3% with *G. morsitans* (e = 4e^−04^), and 27.3% with *Phlebotomus papatasi* (e = 2e^−10^) and *Lutzomia longipalpis* (e = 3e^−11^). The SG5 presented 27.1% identity with *Ae. Aegypti* (e = 1e-30) and 24.3% with *Cx. quiquefasciatus* (e = 1e-34). The specificity of these proteins as biomarkers of exposure to the *Anopheles* genus will have to be verified and, for example, an animal model of exposure could be used. The Ab response against these proteins, or peptides derived from them, can be assessed in rabbits exclusively bitten by *Ae. albopictus*, *Ae. aegypti* or *Cx. quiquefasciatus*. However, the use of the peptides from these proteins is another approach and an opportunity to decrease the possible immune cross-reactivity.

**Table 2:** **Identification/selection of candidate proteins as biomarkers of** ***Anopheles*** **infective bites**

**Protein identification**	**Accession number (UniProtKB/TrEMBL)**	**Spot**	**Fold**	**Mascot score**	**Sequence coverage (%)**	**Peptide number**	**Molecular mass (kDA)**	**pI**
gSG6 ^SP^	Q9BIH5_ANOGA	794	1.8	14	45.22	14	13.1	5.49
gSG1b ^SP^	Q9BIH6_ANOGA	1197	1.6	59	46.23	59	43.6	7.58
Long form D7 ^SP^	Q7PJ76_ANOGA	1144	1.4	15	40.19	15	35.6	5.90
SG5 ^SP^	Q9BIH7_ANOGA	1228	2.0	15	29.22	15	38.2	6.47
		1229	1.6	14	29.52	14		
TRIO protein ^SP^	Q8WR22_ANOGA	819	1.4	12	31.46	12	43.7	6.46
		1228	2.0	21	36.83	21		
		1229	1.6	15	29.41	15		
		1239	1.4	25	40.15	25		

SP, signal peptide.

### Peptide design and antigenicity of selected peptides as potential candidate biomarkers

Aiming to optimize *Anopheles* specificity and to circumvent the limitations in the production of recombinant protein and batch-to-batch variations, peptides from the four previously selected proteins were designed (the gSG1b, long form D7, SG5, TRIO proteins) so as to assess their antigenic properties.

Bioinformatic tools were used to predict potential epitopes for each protein. The comparison of data generated by the four algorithms defined two peptides for gSG1b (gSG1b-P1 and gSG1b-P2), seven peptides for long form D7 (D7-P1 to D7-P7), five peptides for SG5 (SG5-P1 to SG5-P5) and two peptides for the TRIO protein (TRIO-P1 and TRIO-P2), 18–27 amino acid residues long (Table 3). All these peptides were aligned using the Blastp program in Vectorbase to search for similarities with other hematophagous arthropods and the Blastp program in UniProtKB to search for similarities with human infectious organisms, to avoid immune cross-reactivity. No relevant identity was found, as indicated by the low scores observed (few amino acids (aa) consecutively matched and high e-value, i.e., e > 0.03) (Table 4). This analysis showed that all peptides selected possessed a high specificity for the *Anopheles* species.

**Table 3:** **Peptide design of candidate proteins**

**Protein identification**	**Accession number (UniProtKB/TrEMBL)**	**Candidate peptide biomarker**	**Peptide sequence**
gSG6^SP^	Q9BIH5_ANOGA	gSG6-P1	EKVWVDRDNVYCGHLDCTRVATFK
		gSG6-P2	ATFKGERFCTLCDTRHFCECKETREPL
Long form D7^SP^	Q7PJ76_ANOGA	D7-P1	FKALDPEEAWYVYERCHEDHLPS
		D7-P2	DHLPSGPNRETYLKTWKFWK
		D7-P3	GLQMYDEKTNTFKPETVPVQHEAYK
		D7-P4	SRKIYHGTVDSVAKIYEAKPEIKKQ
		D7-P5	NKSDLEPEVRSVLASCTGTQAYDYY
		D7-P6	CTGTQAYDYYSCLLNSPVKEDFRN
		D7-P7	GKVYEGPEKVKEELKKLNY
SG5^SP^	Q9BIH7_ANOGA	SG5-P1	GSLDPLDEEDIRTEQPTSCV
		SG5-P2	VLVSIKSRMMAYTNDAVAKFEHL
		SG5-P3	EECHDKLADHLAEQRREIDAAQ
		SG5-P4	AEQRREIDAAQQLMGEPYRKMDG
		SG5-P5	RRQLMKQNEREVVEKSKS
TRIO protein^SP^	Q8WR22_ANOGA	TRIO-P1	PLTCIRWRSQNPASPAGSLGGKDVV
		TRIO-P2	LGGKDVVSKIDAAMANFKTLF
gSG1b^SP^	Q9BIH6_ANOGA	gSG1b-P1	FEVCLPEIRKDPATAGLVTEV
		gSG1b-P2	KKHMVASKDYESYLGALFAADA

SP, signal peptide.

**Table 4:** **Sequence similarities of peptides with other hematophagous arthropods or human infectious organisms**

**Candidat peptide biomarker**	**Identity (amino acid consecutive, e-value)**							
	***Culex quinquefasciatus***	***Aedes aegypti***	***Glossina morsitans***	***Trichomonas vaginalis***	***Leptospira weilii***	***Cronobacter sakazakii***	***Rhodnius prolixus***	***Trypanosoma cruzi***
D7-P1	4aa, e = 0.036	4aa, e = 0.3						
D7-P2		4aa, e = 0.62						
D7-P3	3aa, e = 0.13			6aa, e = 3.1				
D7-P5			3aa, e = 0.23					
D7-P6	3aa, e = 0.23	2aa, e = 0.046						
D7-P7					6aa, e = 0.5			
SG5-P2						8aa, e = 6.7		
SG5-P4							3aa, e = 0.25	
SG5-P5								3aa, e = 9

**Figure 4:**
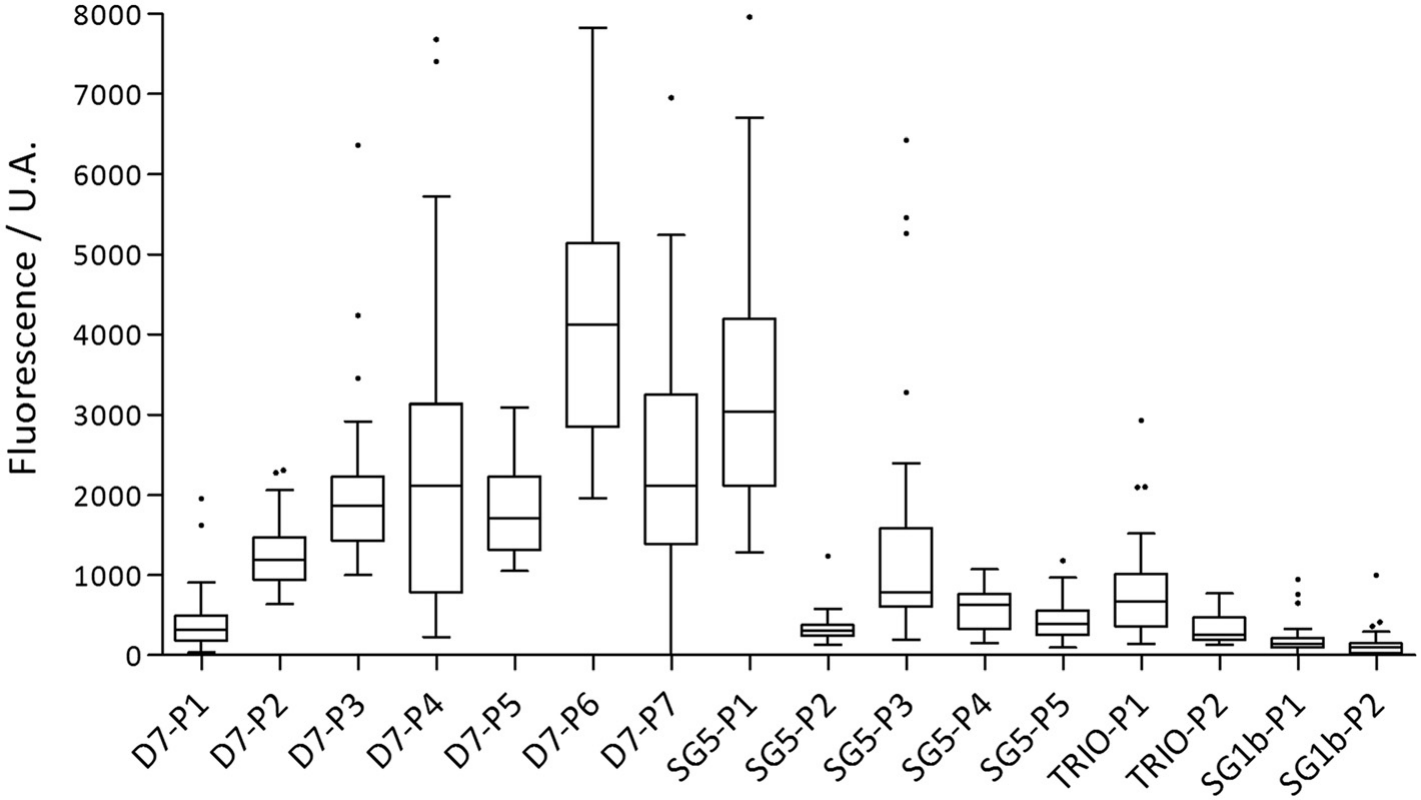
**IgG Ab response according to the different peptides.** The IgG antibody level was evaluated in a sample of children living in Senegal exposed to *Anopheles* bites. Box plots display the median value, 25th and 75th percentiles. Whiskers represent 5 to 95 percentiles and dots the outliers.

The antigenicity of these peptides was assessed using an epitope-mapping approach, with serum from children (*n* = 42) known to be exposed to *Anopheles* bites (Figure 4). All peptides appeared to be antigenic, but different levels of antigenicity between them were observed. The D7-P1, SG5-P2, SG5-P3, SG5-P4, SG5-P5, TRIO-P1, TRIO-P2, SG1b-P1 and SG1b-P2 peptides seemed to have a lower antigenicity than the D7-P2, D7-P3, D7-P4, D7-P5, D7-P6, D7-P7 and SG5-P1 peptides. A high antigenicity could be one of the pertinent criteria for the identification of specific biomarkers of infective bites, but the most important criterion is clearly that this biomarker can differentiate *Anopheles* non-infective and infective bites. Moreover, the level of the specific Ab response observed for each peptide was, for instance, scattered ranging from 2136 to 7136 fluorescence/U.A. for D7-P6 peptide and from 223.5 to 2933.5 fluorescence/U.A. for TRIO-P1 peptide. We cannot exclude that a history of human exposure to *Anopheles* and the immune status of these individuals could greatly contribute to the heterogeneity of the Ab response. The next step of this work will be to evaluate the Ab response to all these peptides on a large scale to compare their antigenicity between individuals infected by *P. falciparum* (individuals previously bitten by infected bites) and individuals exposed to *Anopheles* bites and identified as not infected. However, the fold over-expression of these proteins could also play a role in this differentiation. The up-regulation of the selected proteins varies between 1.4 to 2.0 fold, and future studies are needed to evaluate whether this over-expression is sufficient to differentiate infective and non-infective bites. Combination of different overexpressed peptides (gSG6-P1, gSG6-P2, gSG1b-P1 and gSG1b-P2, D7-P1 to D7-P7, SG5-P1 to SG5-P5, TRIO-P1 and TRIO-P2) could increase the specificity of Ab responses to infective bites. This strategy could also reduce the heterogeneity of Ab responses observed in this study. Future immunological evaluation of these peptides should be conducted using well documented cohorts either for parasitological and immunological status but also using entomological data to validate this approach.

## Conclusion

The present study has provided results on the effect of the presence of the wild *P. falciparum* parasite on the expression of proteins in *An. gambiae* salivary glands. The parasite up- and down-regulates sialome expression, but also seems to induce post-translational modifications. This study and further studies will provide key elements to understand how the insect cells act to protect themselves against infection and how *P. falciparum* manipulates the cellular machinery of the salivary glands and the behavior of *Anopheles* mosquitoes.

In this study, five salivary proteins – gSG6, gSG1b, long form D7, SG5 and TRIO – were selected as potential candidate biomarkers of exposure to *Anopheles* infective bites in order to evaluate the risk of malaria transmission. *Anopheles*-specific and immunogenic peptides were designed from these proteins *in silico*: gSG1b-P1 and gSG1b-P2, D7-P1 to D7-P7, SG5-P1 to SG5-P5, TRIO-P1 and TRIO-P2. Their immunogenicity was tested and validated using blood from children exposed to *Anopheles* bites. These results are the first step toward the development of a biomarker of exposure to *Anopheles* infective bites. This tool is essential to evaluate the malaria transmission in areas of low transmission such as urban settings, highlands and areas where the *P. falciparum* transmission has been tackled by malaria control strategies. The next step will be to check whether all these peptides, in addition to gSG6-P1 and gSG6-P2, can differentiate *An. gambiae*-non-infective from -infective bites.

## Additional file

Additional file 1:
**Identification of Plasmodium falciparum proteins.**

## Abbreviations

Ab: Antibody
WSE: Whole saliva extract
LLIN: Long lasting insecticide-treated nets
IRS: Indoor residual spraying
CHIKV: Chikungunya virus
DENV: Dengue virus
2D-DIGE: Bidimensional differential gel electrophoresis
SGE: Salivary gland extract
GST: Glutathione S transferase
aa: Amino acid
